# The interplay between unexpected events and behavior in the development of explicit knowledge in implicit sequence learning

**DOI:** 10.1007/s00426-021-01630-2

**Published:** 2021-12-24

**Authors:** Clarissa Lustig, Sarah Esser, Hilde Haider

**Affiliations:** Department of Psychology, University of Cologne, Richard-Strauss-Str. 2, 50931 Cologne, Germany

## Abstract

Some studies in implicit learning investigate the mechanisms by which implicitly acquired knowledge (e.g., learning a sequence of responses) becomes consciously aware. It has been suggested that unexpected changes in the own behavior can trigger search processes, of which the outcome then becomes aware. A consistent empirical finding is that participants who develop explicit knowledge show a sudden decrease in reaction times, when responding to sequential events. This so called RT-drop might indicate the point of time when explicit knowledge occurs. We investigated whether an RT-drop is a precursor for the development of explicit knowledge or the consequence of explicit knowledge. To answer this question, we manipulated in a serial reaction time task the timing of long and short stimulus-onset asynchronies (SOA). For some participants, the different SOAs were presented in blocks of either long or short SOAs, while for others, the SOAs changed randomly. We expected the participants who were given a blocked presentation to express an RT-drop because of the predictable timing. In contrast, randomly changing SOAs should hamper the expression of an RT-drop. We found that more participants in the blocked-SOA condition than in the random-SOA condition showed an RT-drop. Furthermore, the amount of explicit knowledge did not differ between the two conditions. The findings suggest that the RT-drop does not seem to be a presupposition to develop explicit knowledge. Rather, it seems that the RT-drop indicates a behavioral strategy shift as a consequence of explicit knowledge.

## Introduction

Implicit learning is a fundamental process in humans. It is said to occur without any intention to learn and, albeit debatable (Shanks, 2005), to lead to knowledge usually not consciously accessible. It enables humans to adapt to regularities inherent in the environment. Examples of implicit learning processes are learning the mother tongue or social behavior in children (Cleeremans, 2008, 2011).

One frequently used paradigm to investigate implicit learning is the serial reaction time task (SRTT; Nissen & Bullemer, 1987). In this task, the participants see a number of marked locations on the screen (often four or six locations) which are mapped to spatially corresponding keys. In every trial, an asterisk appears at one of the locations and the participants’ task is to press the corresponding key as fast as possible. Unbeknownst to the participants, the positions of the asterisk and thus the to-be-pressed keys follow a regular sequence. To assess implicit learning, the regular sequence is replaced by a random sequence after several blocks of training and is re-introduced thereafter. The usual finding is that the reaction times (RTs) increase when the regular sequence is replaced by a random sequence and decrease when the regular sequence is re-introduced. Despite of this performance decrement, the participants are often unable to name the sequence. This led many researchers to conclude that learning in the SRTT is implicit and does not necessarily lead to explicit knowledge (Nissen & Bullemer, 1987). A common finding is, however, that a few participants indeed show at least some explicit knowledge about the practiced sequence.

Given that there are participants who acquire explicit knowledge in an implicit learning situation, an important question arises: Which cognitive mechanisms underlie this interplay between implicit and explicit knowledge? One possibility to account for the acquisition of conscious knowledge in an implicit learning situation is to assume that learning leads to a gradual strengthening of the acquired representations (Cleeremans & Jiménez, 2002).

A different proposal is made by the Unexpected-Event Hypothesis (UEH; Esser et al., 2021; Frensch et al., 2003). The central assumption of the UEH is that, during SRTT training, participants acquire unconscious knowledge about the sequence inherent in the task. This unconscious knowledge is not assumed to become conscious by mere strengthening of the underlying representation. Rather, implicit knowledge is assumed to lead to consciously perceivable behavioral changes, such as the feeling of fluency or the urge to respond even though the stimulus is not entirely processed. Since implicit knowledge is unconscious, such changes are likely to be unexpected and might violate participants’ expectations about their task performance (Koriat, 2012; Whittlesea, 2004). Therefore, if a participant detects such an unexpected behavioral change, it will likely trigger an explicit search process for the causes of this experienced change. This search processes in turn might lead to the detection of the regular sequence and thus to conscious sequence knowledge. Thus, the central assumption of the UEH is that an explicit representation of the sequence results from a second inferential process, rather than only from strengthening the unconscious representation. A second assumption of the UEH is derived from the Global Workspace theory (Dehaene & Naccache, 2001; Marti & Dehaene, 2017). According to the Global Workspace theory, a conscious representation results from bottom-up strengthening, on the one hand, and, on the other hand, from a neurological “ignition”, a sudden, strong top–down activation of a vast variety of cortical and subcortical regions (Dehaene & Naccache, 2001; Del Cul et al., 2009). Taking this into account, it is assumed in the UEH that the insight into the sequence occurs abruptly in an all-or-none manner (Esser et al., 2021).

The assumptions of the UEH fit well with similar assumptions that are proposed by the Event Segmentation Theory (EST; Zacks et al., 2007) about the human experience of events. According to the EST, active neuronal processing causes memory representations of events that lead to a sense of the current moment and in sum build the content of working memory (the event model). These memory representations enhance perception as well as prediction of future events. Analogous to the UEH, Zacks et al. assume that processing is always monitored by error detection processes. If an unexpected event occurs that does not fit with the predictions, a prediction error is detected. Different to the UEH, the EST does not assume that search processes are started as a consequence of the detected prediction error. In contrast, the EST assumes that the current event model will be updated by building a new set of memory representations. Thus, the EST describes more globally how changes in the perception of events might occur, whereas the UEH focuses more on the special situation of insight processes in implicit learning.

In the last years, the UEH gained support from various studies. For example, Haider and Frensch (2005, 2009) tested the role of premature responses (responses that are entered before the stimulus is entirely encoded) as an instantiation of unexpected events. Their findings suggest that increasing the probability of such premature responses either through long RSIs or through computer-generated premature responses led to more explicit knowledge.

Rünger and Frensch (2008) generated unexpected events by disrupting the sequence within an SRTT experiment. After some training blocks, the participants were transferred to either a different sequence, a random sequence, or a dual task situation. The findings revealed that only the interruption through a different sequence led to increased amounts of explicit knowledge. They concluded that merely interrupting the participants, as was the case when a dual task situation was introduced, was not sufficient to increase the amount of explicit knowledge. Instead, only interruptions that do not disrupt search processes lead to higher amounts of reportable knowledge.

Recently, Esser and Haider (2017) provided further evidence that mere strengthening of the underlying sequence representation is not sufficient to explain the emergence of explicit sequence knowledge. They trained participants with a sequence interspersed with deviants. These deviants were either presented in a blocked or a random order. Even though the number of sequence trials was identical in both conditions, the participants in the blocked order condition developed more explicit knowledge than the participants in the random order condition. According to the UEH, the participants in the blocked order condition could experience an unexpected variation in fluency while performing the task. This violation of expectancies led to attribution processes and thus to the development of explicit knowledge. By contrast, the participants in the random order condition might have experienced the task as constantly fluent and therefore might not have experienced a violation of their expectations.

In sum, these studies fit with the UEH by showing that introducing discrepancies between the expected and the actual behavior increases the amount of explicit knowledge. Furthermore, most of these findings cannot be explained by mere strengthening of the implicit knowledge because the amount of training was kept constant in all mentioned studies.

Another line of empirical studies focuses on the second assumption of the UEH that explicit knowledge occurs abruptly in an all-or-none manner. These studies found high correlations between the ability to report the entire sequence in a post-experimental knowledge test, and an abruptly occurring decrease in RTs during the training phase (Frensch et al., 2003; Haider & Rose, 2007). This sudden decrease of RTs, the so called RT-drop, is thought to indicate a qualitative shift of the processing strategy from stimulus-driven to top–down-driven processing (Haider & Frensch, 2005; Haider et al., 2005, 2011; Rose et al., 2010; Wessel et al., 2012). If participants explicitly know the entire sequence, they do not need to process the imperative stimulus to produce the required response (Haider et al., 2011; Koch, 2007; Tubau & Lopéz-Moliner, 2004; Tubau et al., 2007).

Going beyond the findings of behavioral experiments, Rose et al. (2010) as well as Wessel et al. (2012) conducted fMRI and EEG studies within a sequence learning paradigm. They analyzed the data time locked to the individual appearance of the RT-drop. The findings revealed that the RT-drop was accompanied by a strong increase in neuronal activity (fMRI) and in high-frequency coupling (EEG) between distant brain areas (prefrontal, parietal, and occipital). Similar results came from Schuck et al. (2015), who investigated strategy shifts in a rather simple task and found also that the participants abruptly sped up responding when they became aware of a more efficient strategy than that they had used before. Right before this changing point, Rose et al. (2010) as well as Schuck et al. (2015) found increased activity in the medial prefrontal cortex (for similar results see Lawson et al., 2017). These activation patterns are associated with the transition from unconscious to conscious representations (Del Cul et al., 2009).

Given the results from the neurophysiological studies as well as the high correlations between the occurrence of RT-drops and verbal reports, it seems a reasonable conclusion that the RT-drop might define the point in time when explicit knowledge occurs. The findings suggest that, at this point in time, the participants change their processing strategy from stimulus-driven to top–down (Haider et al., 2011; Schuck et al., 2015; Tubau & Lopéz-Moliner, 2004; Tubau et al., 2007; Wessel et al., 2012).

However, the exact role of the RT-drop for the development of conscious knowledge about the sequence has been unclear, yet. On the one hand, the RT-drops may occur as a side effect of having developed a consciously accessible representation of the sequence. On the other hand, they might be a precursor for conscious awareness about the sequence. Shedding light on this issue will help to better understand how explicit knowledge and performance are linked. Furthermore, knowing about the exact role of RT-drops might provide some methodological implications for further investigations.

## Overview of the Current Study

The goal of the current study was to investigate the specific role of RT-drops for the development of explicit knowledge. In the above mentioned studies, the occurrence of the RT-drop was highly correlated with the ability to verbally report the entire sequence (Haider & Frensch, 2005; Haider & Rose, 2007; Haider et al., 2011; Wessel et al., 2012). Together with the neuroimaging studies, this suggests that the RT-drop might indicate the point in time when an explicit representation of a given rule in an implicit learning paradigm arises. However, it has never been tested experimentally, whether the RT-drop really indicates the transition from implicit to explicit knowledge or whether it only indicates the strategy-change that results from having acquired explicit knowledge.

If the RT-drops were a precursor for the development of a conscious representation of the sequence, preventing the participants from changing their strategy should reduce the amount of verbally reportable knowledge (Cleeremans, 2006; Haider & Frensch, 2009). By contrast, if an RT-drop indicates the translation of an otherwise acquired explicit representation into performance, preventing participants from changing their strategy should not have any effect on the development of explicit knowledge per se but only on the behavioral expression of what has been learned explicitly.

For this purpose, we conducted an SRTT with a first-order 6-element motor sequence and manipulated the likelihood that an RT-drop would occur. This was done in two ways: First, we used two different stimulus-onset asynchronies (SOAs) between the presented locations on the screen (the trial onset) and the appearance of the target stimulus, a short (200 ms) and a long (800 ms) one. These two SOAs were presented in either a blocked order (a series of 60 trials of each of the respective SOAs in each training block; blocked-SOA condition), or randomly (random-SOA condition). Second, responses were only valid if they were entered after the target onset to hinder participants from responding prematurely (Haider & Frensch, 2009). Furthermore, we used a rather short response window of 500 ms starting with the target onset. This was done to force the participants to wait—in particular, when the SOA was long—for the appearance of the target and then to immediately enter their response. Consequently, with increasing practice, these long SOAs should lead to a strong urge to respond right before the stimulus appears (see, e.g., Grosjean et al., 2001). If the stimulus then occurs and corresponds to the anticipated response, this should come along with a surprise. In an implicit learning situation, the participants are not informed about the existence of a sequence. Thus, knowing the response in advance to the presentation of a target should likely violate the participants’ expectations. This surprising experience together with not being aware about the cause (the sequence), should, in turn, trigger explicit search processes in both conditions.

Therefore, our manipulations should not affect the experience of an unexpected event per se because all participants should experience a possibly surprising urge to respond after a long SOA. Rather, the manipulation of the SOA-order should affect the likelihood that the participants will detach their responding from the appearance of the target and thus will rely their performance on only the explicit sequence representation (Gaschler et al., 2019; Shin, 2008; Willingham et al., 1997). In the blocked-SOA condition, the two different SOAs are predictable such that the participants could anticipate the point in time when the target will occur. In contrast, the SOAs in the random-SOA condition vary accidentally making it rather impossible to anticipate the exact stimulus-onset in a given trial (e.g., Niemi & Näätänen, 1981). Therefore, the participants in this latter condition are forced to respond after the target has been presented. This should reduce the likelihood that the participants will rely their performance on their explicit representation of the sequence, and thus, will not show an RT-drop (the switch from a data-driven to a top–down processing strategy). If the RT-drops were a precursor for conscious awareness about the sequence to develop, the amount of explicit knowledge within this condition should be reduced.

## Methods

### Participants

One hundred forty one volunteers (81 men) with a mean age of 28.29 years (SD = 9.74) participated in the experiment either for course credit or for payment (4€, respectively, 3.75£). These volunteers were acquired online among students from the University of Cologne (*N* = 23) as well as German participants recruited on the online platform Prolific Academic (https://www.prolific.co; *N* = 118). The participants were randomly assigned to the blocked-SOA (*N* = 67) and random-SOA (*N* = 74) conditions.

The data from participants were excluded if their mean accuracy rate was lower than 60%. We used a liberal accuracy criterion because the fixed response window had increased the likelihood to miss a response. Thirteen participants in the blocked-SOA (with *M* = 34% misses (SD = 18%) and *M* = 30% errors (SD = 12%)) and twelve participants in the random-SOA conditions [with *M* = 24% misses (SD = 19%) and *M* = 36% errors (SD = 20%)] were excluded.^1^

### Materials

The experiment was programmed with Psychopy and uploaded as JavaScript in Pavlovia (https://pavlovia.org/). We used a variation of the standard SRTT from Nissen and Bullemer (1987). In our version of the SRTT, six black response squares (2 × 2 cm) appeared on a horizontal line in the middle of the screen. The response squares were spatially mapped to the six response keys Y, X, C, B, N, and M on a German QWERTZ-keyboard. In every trial, the picture of a mouse appeared as the target in one of the response squares and the participants were instructed to press the corresponding key as fast as possible to catch the mouse. The locations of the target and thus of the response keys followed a repeated 6-element first-order sequence (526341).^2^ After each trial, the participants were informed whether they responded too early or too late (“fail!”), whether their response was incorrect (“error!”) or whether they responded correctly and in the accurate timing (“correct!”). At the end of the experiment, the participants were interviewed via socscisurvey (SoSci Survey GmbH. https://www.soscisurvey.de).

### Procedure

The experiment started with computer-based instructions. Then, the participants in both conditions completed the SRTT that consisted of 6 blocks with 120 trials, each. After each block, the participants were allowed to take a short break.

Each trial started with the presentation of the six response squares on the screen. After either a short (200 ms) or a long SOA (800 ms), the target stimulus appeared at one of the six positions for 150 ms. Starting with the target onset, the participants had a response window of 500 ms to press the respective key. Each trial ended with the feedback about the actual performance (fail, error, or correct), which was displayed for 300 ms. After the feedback, the next trial started immediately with the presentation of the six response squares.

The participants in both conditions received 60 short and 60 long SOAs in each block. In the blocked-SOA condition, the participants started with the 60 short SOAs followed by the 60 long SOAs. For the participants in the random-SOA condition, the SOAs changed unpredictably from trial to trial.

After having finished the SRTT training, the participants completed 150 trials of a post-decision wagering task (Persaud et al., 2007) to assess their sequence knowledge. The wagering task is identical to the SRTT with the only exception that in 36 trials a question mark appears at one of the six positions instead of the target. In these trials, the participants had to guess the position of the next target by pressing the corresponding key. Afterward, they were asked to place a high (50 Cent/Pence) or low (1 Cent/Penny) wager, depending on their confidence in the correctness of their response. The rationale behind this task is that the participants with predominantly implicit sequence knowledge should not be able to maximize their gains and, therefore, the correctness of their responses should not correlate with their wagers (Dienes & Seth, 2010). However, the participants with predominantly explicit knowledge are highly confident in the correctness of their responses and should place more high wagers when responding correctly. In the current experiment, the participants could win a maximum of 3€/2.5£. The task did not have any time restrictions and had a fixed SOA of 400 ms. The participants were informed about the changes in this part of the experiment and about the possibility of the bonus payment. However, they were not informed about the existence of a motor sequence and did not receive any feedback about the correctness of their responses.

Finally, the participants were forwarded to an online survey. Participants were asked whether they had noticed any sequence or whether they thought that there was no system at all. Afterward, the participants were informed that a motor sequence has been built into the task and were asked to reproduce the complete sequence as best as they could.

### RT-drop analysis

The procedure for determining the RT-drop was similar to that of Haider et al. (2011; see, also, Wessel et al., 2012). The authors adapted the method from Haider and Rose (2007; Rose et al., 2010), which was developed to detect the RT-drop in a slightly different task, the number reduction task. The procedure is based on the assumption that sequential knowledge in a SRTT is formed in chunks (Schlaghecken et al., 2000). This means that not all transitions between two succeeding sequence elements drop at the same time. Rather, they may drop one after the other with an individual time delay. Based on this rationale, we initially calculated the RT-drop for each single transition of the sequence.

**Fig. 1:**
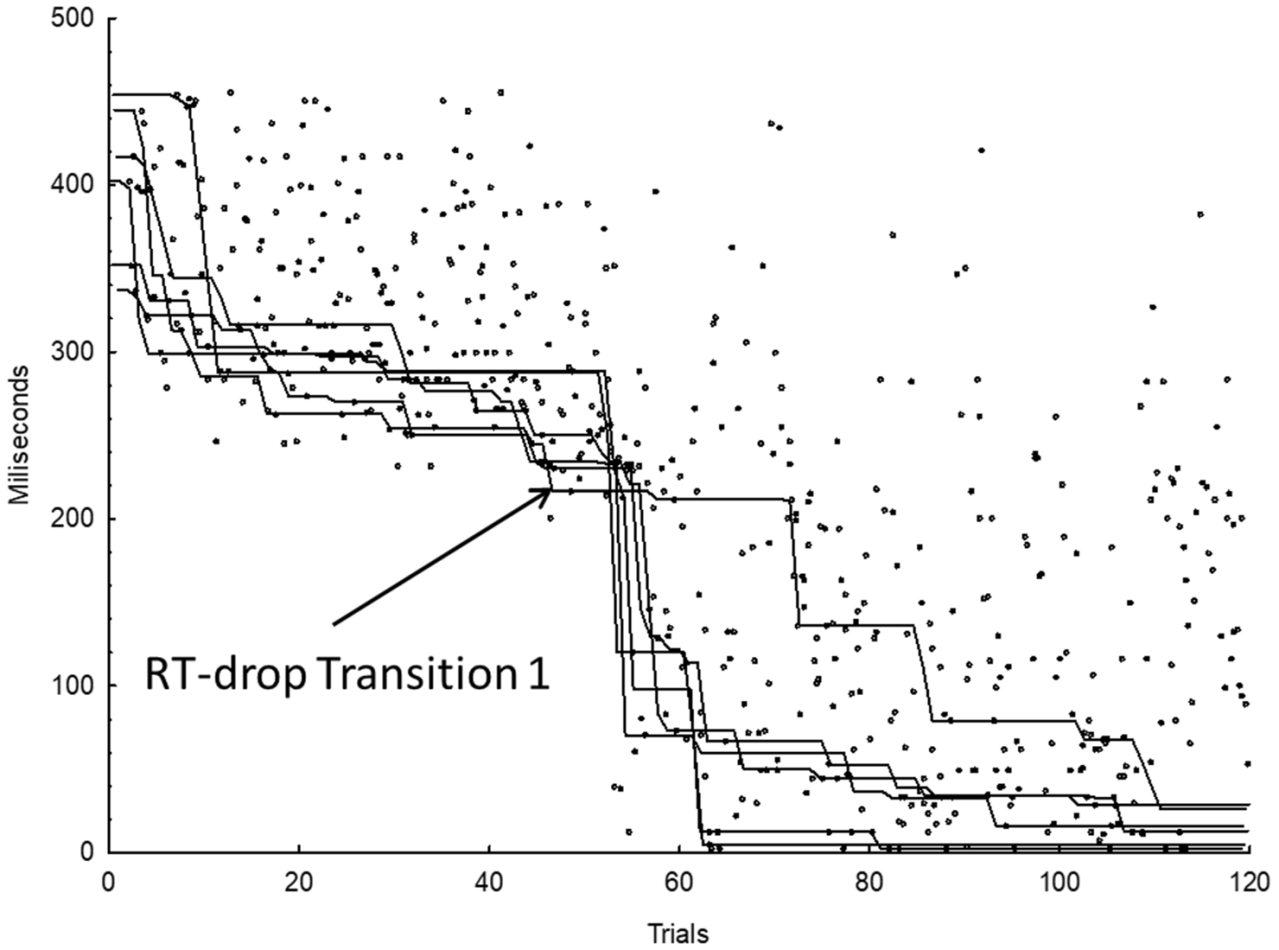
Minimum functions per transition (lines) and raw RTs (dots) as illustration for the RT-drop detection. The detected RT-drop for Transition 1 is marked as an example

Calculation of the RT-drops of each single transition was as follows: First, we removed all erroneous and missing responses and replaced them by the mean of the preceding and the subsequent trials. Second, we computed a median filter per transition with a lag of 3 subsequent repetitions to reduce noise in the data. Third, a minimum function was computed (Haider & Rose, 2007). That is, the value of this function changed only when the current median RT was shorter than the previous one. In all other cases, the value of this function remained unchanged. This produces a monotonously decreasing RT-function for each sequence transition. Fourth, to define the RT-drop for each single SRTT transition, we computed confidence intervals including all remaining transitions (as long as they have no diagnosed RT-drop). For each transition, we defined the point in time when the minimum function of this respective transition dropped below the confidence interval of the combined minimum function of the remaining transitions. If an RT-drop was detected for one transition, no further RT-drop was searched for this single transition. In addition, the RT of the minimum function in the trial immediately before the diagnosed drop of this transition was used to compute the combined minimum functions for the RT-drop detection of the remaining transitions. Fifth, to avoid the detection of too early pseudo-RT decreases due to familiarization with the task, we defined a threshold of 200 ms. Only if the minimum function of one transition fell below the confidence interval of the combined minimum functions of the remaining transitions and below the threshold of 200 ms, it was defined as an RT-drop. Finally, the overall RT-drop for one individual participant was defined as that point in time when at least 4 out of 6 sequence transitions had dropped (Haider et al., 2011). Figure 1 shows an example for the RT-drop detections for the six transitions for one participant.

It is important to note, that using the minimum function to determine the RT-drops within participants should reduce the likelihood that the probably more varying RTs in the random-RSI-order condition affect the detection of RT-drops.

## Results

For each participant and each block, median RTs and mean error rates were computed separately. For the RTs, erroneous trials (7.5% of all data) and missings (8.85% of all data) were excluded. As the response window was restricted, there were no RT-outliers to exclude. For all statistical analyses, an α-level of 5% was adopted. If Mauchly’s test of sphericity reached significance, Greenhouse–Geisser corrected *p *values are reported together with the original degrees of freedom.

### Error rates and latencies in the SRTT

**Fig. 2:**
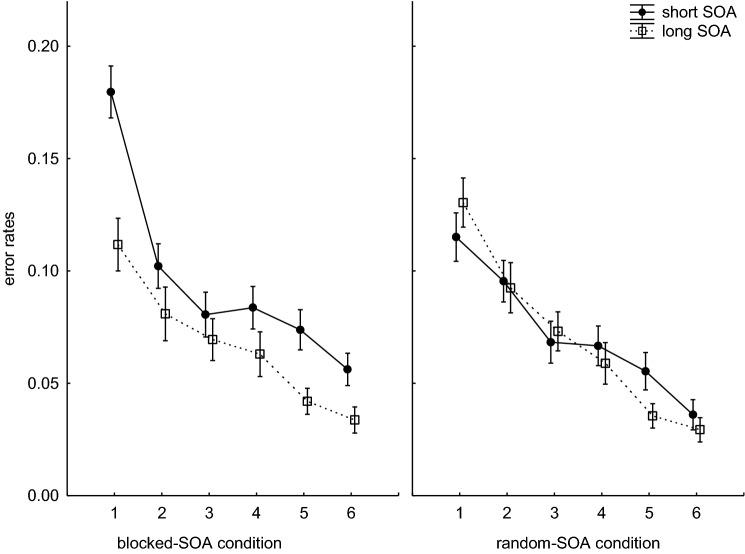
Mean percent error rates per block and SOA. Error bars reflect the standard errors of the means

Figure 2 shows the mean error rates per block and condition separately for long and short SOAs. For the mean error rates as dependent variable, a 2 (SOA-condition: blocked-SOA, random-SOA) × 2 (SOA-length: short, long) × 6 (Block) ANOVA with repeated measures for the last two factors revealed a significant main effect of Block (*F*(5,570) = 73.39, *p* < 0.001, *η*_*p*_^2^ = 0.39). As can be seen from Fig. 2, the error rates in both SOA conditions decreased over time. The main effects of SOA-condition (*F*(1,114) = 1.15, *p* = 0.285, *η*_*p*_^2^ = 0.00) as well as the interaction between SOA-condition and Block (*F*(5,570) = 1.17, *p* = 0.319, *η*_*p*_^2^ = 0.01) were not significant. The main effect of SOA-length (*F*(1,114) = 26.59, *p* < 0.001, *η*_*p*_^2^ = 0.18) and also the two-way interactions between SOA-condition and SOA-length (*F*(1,114) = 17.93, *p* < 0.001, *η*_*p*_^2^ = 0.13), between SOA-length and Block (*F*(5,570) = 3.15, *p* = 0.014, *η*_*p*_^2^ = 0.02), as well as the triple interaction (*F*(5,570) = 7.94, *p* < 0.001, *η*_*p*_^2^ = 0.06) were significant. Figure 2 shows that the participants made more errors when the SOA was short than when it was long. This difference was more pronounced for the participants in the blocked-SOA condition.

Separate post hoc tests for each SOA-condition show that the difference in error rates for long and short SOAs was significant for the participants in the blocked-SOA condition (short SOA: *M* = 9.59%; SD = 6.49%; long SOA: *M* = 6.67%; SD = 5.40%; *t*(53) = 6.36, *p* < 0.001), but not for the participants in the random-SOA condition (short SOA: *M* = 7.28%; SD = 4.02%; long SOA: *M* = 6.99%; SD = 5.10%; *t*(61) = 0.68, *p* = 0.498). Furthermore, the two SOA conditions did not differ with regard to the long SOAs (*t*(114) = 0.32, *p* = 0.746). However, considering only the short SOAs, the error rates were significantly higher in the blocked-SOA condition than in the random-SOA condition (*t*(114) = 2.34, *p* = 0.020). This was particularly the case at the beginning of training.

**Fig. 3:**
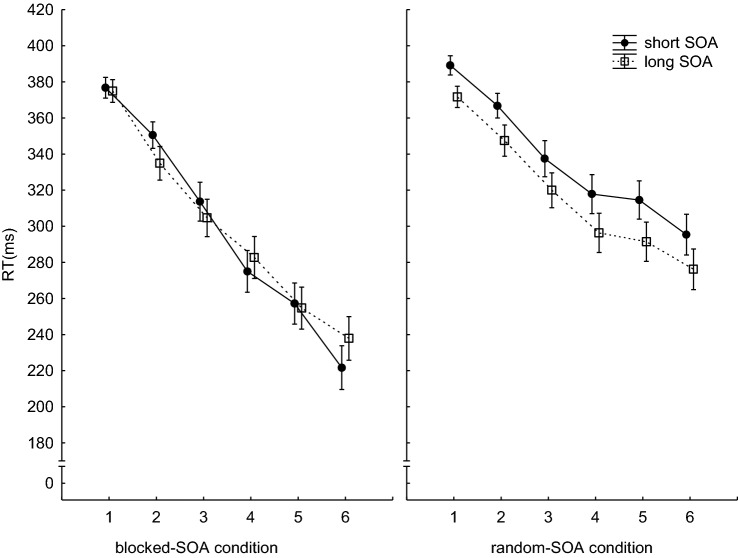
Mean RTs per block and SOA. Error bars reflect the standard errors of the means

The mean RTs are shown in Fig. 3.^3^ The 2 (SOA-condition: blocked-SOA, random-SOA) × 2 (SOA-length: short, long) × 6 (Block) ANOVA with RT as dependent variable showed a significant main effect of Block (*F*(5,570) = 164.34, *p* < 0.001, *η*_*p*_^2^ = 0.59). Here, the main effects of SOA-condition (*F*(1,114) = 5.61, *p* = 0.019, *η*_*p*_^2^ = 0.04) as well as the SOA-condition × Block interaction (*F*(5,570) = 7.85, *p* < 0.001, *η*_*p*_^2^ = 0.06) were significant. As can be seen from Fig. 3, the RTs of the participants in both SOA conditions decreased over time. Furthermore, the participants in the blocked-SOA condition responded overall faster than the participants in the random-SOA condition. This RT-difference increased with training. In addition, the main effect of SOA-length (*F*(1,114) = 24.15, *p* < 0.001, *η*_*p*_^2^ = 0.17), the two-way interactions between SOA-condition and SOA-length (*F*(1,114) = 20.42, *p* = 0.012, *η*_*p*_^2^ = 0.15), and between SOA-length and Block (*F*(5,570) = 3.10, *p* = 0.008, *η*_*p*_^2^ = 0.02), as well as the triple interaction (*F*(5,570) = 3.64, *p* = 0.002, *η*_*p*_^2^ = 0.03) were significant. As the data in Fig. 3 suggest, the participants in the blocked-SOA condition did not show any systematic difference between long and short SOAs. However, the participants in the random-SOA condition were significantly faster with the long than with the short SOAs. The interaction between SOA-length and block and the triple interaction did not indicate a clear trend.

To further investigate the differences between the SOA conditions, we also conducted separate 2 (SOA-length: short, long) × 6 (Block) ANOVA for the two SOA conditions. For the blocked-SOA condition, the main effect of Block (*F*(5,265) = 87.17, *p* < 0.001, *η*_*p*_^2^ = 0.62) as well the interaction between SOA and Block (*F*(5,265) = 3.31, *p* = 0.010, *η*_*p*_^2^ = 0.05) reached significance (for SOA (*F*(1,53) < 1). For the random-SOA condition, the main effects of SOA-length (*F*(1,61) = 104.45, *p* < 0.001, *η*_*p*_^2^ = 0.63) and for Block (*F*(5,305) = 74.57, *p* < 0.001, *η*_*p*_^2^ = 0.55) were significant, while the SOA-length x Block interaction was not (*F*(5,305) < 1). The data in Fig. 3 show that, for the random-SOA condition, the difference between the long and short RSI lengths remains rather constant over all blocks.

To sum up, our manipulation of the order of the SOAs affected both, the error rates and the RTs. In the blocked-SOA condition, the participants reacted faster than the participants in the random-SOA condition. However, optimizing response speed seems to come at costs, since error rates for the short SOA trials were higher in the blocked-SOA condition.

In contrast, in the random-SOA condition, the pattern of results suggests slower responses for short than for long SOA trials. Whether this was accompanied by an increased accuracy is ambiguous. On the one hand, the error rates did not systematically differ between the two SOA types. On the other hand, the participants in the random-SOA condition made fewer errors than the participants in the blocked-SOA condition. Since a baseline is missing, it remains unclear whether the error rate was increased in the blocked-SOA condition or reduced in the random-SOA condition.

Overall, the error rates and the RT findings together suggest that our SOA manipulation was successful. The slower responses in the random-SOA compared to the blocked-SOA condition suggest that the exact timing of the responses was more difficult in the former SOA condition. However, a not mutually exclusive explanation for the overall shorter RTs in the blocked-SOA condition might be that more participants exhibited an RT-drop in this condition than in the random-SOA condition, since these participants were better able to adapt and optimize their performance according to the respective SOAs. Thus, the next important question concerns the effect of our SOA manipulation on the rate of RT-drops.

### RT-drop

Based on the procedure described in the methods section, we conducted the RT-drop analyses for each individual participant. In the blocked-SOA condition, 39 participants (72%) showed an RT-drop as defined above, whereas only 26 participants (41%) in the random-SOA condition did so. This difference between the two conditions was significant (*χ*^2^(1) = 10.75; *p* = 0.001, *φ* = 0.30). Thus, our manipulation of the SOA arrangement not only affected RTs and error rates, but also the probability that the participants in the random-SOA condition exhibited an RT-drop. On the basis of these findings, we investigated our main hypothesis concerning the relationship between RT-drops and explicit knowledge.

### Post-decision wagering task (PDWT)

In the PDWT, as the main test for explicit knowledge, only the wagering trials were considered. For these trials, we computed percent correct responses as a measurement of overall acquired sequence knowledge. In addition, we also computed the percent correct responses under high (correct|high) versus low wagers (correct|low) as a measurement of the subjective certainty to have responded correctly and thus as a measure of explicit knowledge. The results are presented in Table 1.

**Table 1:** Percent correct responses and certainty judgements for the blocked-SOA and random-SOA condition. The respective standard deviations are presented in brackets (*C*|*H* refers to correct|high; *C*|*L* to correct|low)

SOA-condition	Correct (%)	*C*|*H*	*C*|*L*	*N* total	*N* explicit (%)
Blocked-SOA	76.28 (24.19)	79.30 (28.00)	49.02 (33.09)	54	37 (68.51)
Random-SOA	75.31 (25.97)	77.49 (28.97)	46.41 (35.86)	62	38 (61.29)

Concerning the percent correct responses, the participants in both SOA conditions had more sequence knowledge than expected by chance (chance level: 20%; blocked-SOA condition: *t*(53) = 7.21, *p* < 0.001; random-SOA condition: *t*(61) = 7.03, *p* < 0.001). More important, the two SOA conditions did not differ with regard to their amount of correct responses (*t*(114) = 0.20, *p* = 0.835). To further bolster this insignificant difference between the two SOA conditions, we computed a Bayes Analysis with JASP (JASP Team, 2020; Morey & Rouder, 2015) for *t* tests (Rouder et al., 2009). We calculated a Bayes Factor *BF*_*01*_ to test the null hypothesis (H_0_) to be favored over the alternative hypothesis (H_1_). We used the Cauchy distribution as prior distribution with a Cauchy scale of 0.0707 for the calculation of Bayes Factors for *t *tests with JASP (JASP Team, 2020). Our Bayes factor was *BF*_*01*_ = 4.96, which, according to Jeffreys (1939/1961), provides evidence for the null hypothesis.

The certainty judgements served to test the amount of explicit sequence knowledge. A 2 (SOA-condition: blocked-SOA, random-SOA) × 2 (Certainty Judgment: correct|high, correct|low) ANOVA with percent correct responses as dependent variable was conducted. A significant main effect of Certainty Judgment (*F*(1,114) = 61.40, *p* < 0.001, *η*_*p*_^2^ = 0.35) was found. As can be seen in Table 1, the participants in both SOA conditions placed more high than low wagers for correct responses. Importantly, there was again no main effect of SOA-condition (*F*(1,114) = 0.25, *p* = 0.617, *η*_*p*_^2^ = 0.00) and no SOA-condition × Certainty Judgment interaction (*F*(1,114) = 0.01, *p* = 0.919, *η*_*p*_^2^ = 0.00). Thus, the two SOA conditions did not differ regarding their confidence in the correctness of their responses and therefore in their amount of explicit knowledge. Additionally, we computed a Bayes Analysis for ANOVA designs (Rouder et al., 2012) to validate the finding that the two SOA conditions did not differ in the amount of their explicit knowledge. The calculated Bayes Factor was *BF*_*01*_ = 5.72 indicating that the null hypothesis can be favored over the alternative hypothesis.

In addition, we identified those participants who had acquired predominantly explicit sequence knowledge. The participants were classified as possessing predominantly explicit knowledge when they responded correctly in at least 70% of all wagering trials and placed primarily high wagers for correct answers. The number of participants with explicit knowledge in each SOA-condition is shown in Table 1. Again, the two SOA conditions did not differ (*χ*^2^(1) = 0.66; *p* = 0.416, *φ* = 0.075).

In sum, the results of the PDWT show that the participants in both conditions had acquired a comparable amount of sequence knowledge. Furthermore, this knowledge seems to be primarily explicit as the participants placed high wagers on their correct responses and thus, showed a high confidence in the correctness of their responses. Approximately 70% of the participants in each condition were classified as having full explicit knowledge about the motor sequence. Thus, even though there were fewer RT-drops in the random-SOA condition, the amount of explicit knowledge about the sequence did not differ from that of the blocked-SOA condition.

However, two alternative explanations concerning the discrepancy between the amount of explicit knowledge and the occurrence of an RT-drop in the random-SOA condition need to be discussed. First, it might be that our procedure used to diagnose the RT-drops was not a valid method. Second, it is conceivable that the PDWT did not validly assess what the participants knew about the sequence. To handle these considerations, we conducted two post hoc analyses.

### Post hoc analyses

**Fig. 4:**
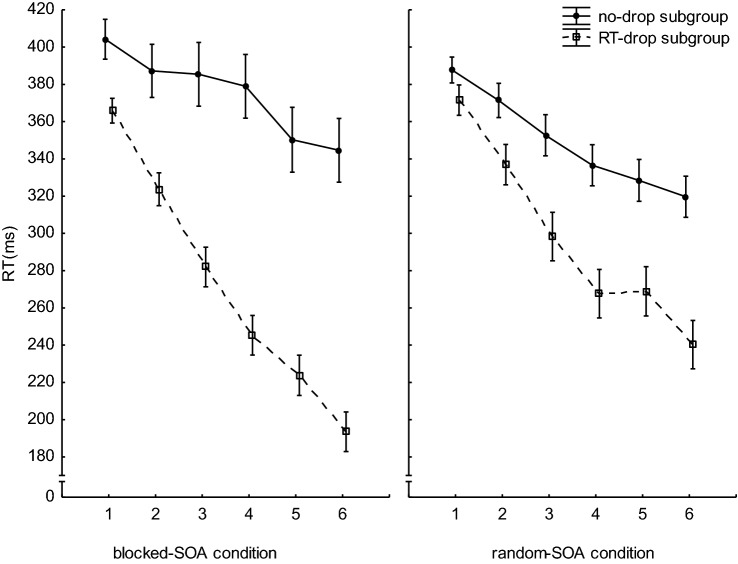
Mean RTs per block for the participants with and without RT-drop. Error bars reflect the standard errors of the means

To validate our RT-drop analysis, we divided both SOA conditions into two subgroups, one with the participants with (drop subgroup) and one with the participants without an RT-drop (no-drop subgroup). In addition, we tested whether the RT-drops were indicative for the amount of explicit knowledge. If our procedure to diagnose the RT-drops was valid, we should find a stronger RT-decrease in both drop subgroups. Furthermore, the drop subgroups should show more explicit knowledge about the sequence. The mean RTs for the first analysis are shown in Fig. 4.

We conducted a 2 (SOA-condition: blocked-SOA, random-SOA) × 2 (Subgroup: RT-drop, no-drop) × 6 (Block) ANOVA with RT as dependent variable and found a significant main effect of Block (*F*(5,560) = 136.31, *p* < 0.001, *η*_*p*_^2^ = 0.54) and of Subgroup (*F*(1,112) = 54.95, *p* < 0.000, *η*_*p*_^2^ = 0.32). The main effects of SOA-condition (*F* < 1) as well as the SOA-condition × Block interaction (*F*(5,560) = 1.91, *p* = 0.090, *η*_*p*_^2^ = 0.01) were not significant. The interactions between Subgroup and Block (*F*(5,560) = 23.46, *p* < 0.001, *η*_*p*_^2^ = 0.17) the interaction between SOA-condition and Subgroup (*F*(1,112) = 5.86, *p* = 0.017, *η*_*p*_^2^ = 0.04) as well as triple interaction (*F*(5,560) = 2.30, *p* = 0.043, *η*_*p*_^2^ = 0.02) were significant. Figure 4 shows that within both SOA conditions, the RTs of the participants in the drop subgroup decreased faster with practice than in the non-drop subgroup. This difference was greater in the blocked-SOA condition than in the random-SOA condition. However, the qualitative picture was rather similar in both SOA conditions. This suggests that the RT-drop analysis reliably identified the participants with an RT-drop in both SOA conditions. It is rather unlikely, that the rate of miss-classified participants in the random-SOA condition is much higher than that in the blocked-SOA condition. If this had been the case, we should have found no significant RT-differences between the two subgroups within the random-SOA condition.

To test whether the RT-drops are indicative for explicit sequence knowledge (Haider & Rose, 2007), we also analyzed, how many participants in the two subgroups (drop, no-drop) within both SOA conditions (blocked-SOA and random-SOA) showed predominantly explicit knowledge? The results are shown in Table 2. In both SOA conditions, approximately 80% of the participants in the drop subgroup possessed predominantly explicit knowledge. A *χ*^2^-test conducted separately for the two SOA conditions confirmed that this rate was significantly higher than that in the two non-drop subgroups (blocked-SOA condition: *χ*^2^(1) = 11.92; *p* = 0.006, *φ* = 0.46; random-SOA condition: *χ*^2^(1) = 7.16; *p* = 0.007, *φ* = 0.33). Only the effect size in the random-SOA condition was smaller than that in the blocked-SOA condition. Thus, in both SOA conditions, the RT-drop seems to be indicative for explicit knowledge to occur.

**Table 2:** Participants with predominantly explicit knowledge in the blocked-SOA and random-SOA condition

Subgroup	SOA-condition			
	Blocked-SOA		Random-SOA	
	*N* total	*N* explicit (%)	*N* total	*N* explicit (%)
Drop	39	32 (82.05)	26	21 (80.77)
No-drop	15	5 (33.33)	36	17 (47.22)

Despite of this difference between the two subgroups, the number of participants with predominantly explicit knowledge in the no-drop subgroup of the random-SOA condition is higher than that in the blocked-SOA condition. This latter finding is in line with our assumption that the SOA manipulation affected the RT-drop rate in the random-SOA condition, but not the development of explicit knowledge.

Taken together, the two post hoc analyses that we conducted to verify our RT-drop analysis revealed that the behavioral patterns of the two subgroups (drop, no-drop) were qualitatively similar within both SOA conditions (blocked-SOA, random-SOA). The main difference between the two SOA conditions was that, probably due to the higher number of participants with RT-drops in the blocked-SOA condition, the performance differences between the two subgroups were more pronounced within this condition. Given these results, it seems justified to assume that our main finding of more RT-drops in the blocked-SOA condition and comparable explicit knowledge in both SOA conditions, was not due to a lesser likelihood to correctly diagnose the RT-drops in the random-SOA condition.

### Post-experimental interview

For our second question, whether the PDWT validly assessed participants’ explicit knowledge, we analyzed the data of our post-experimental interview. For each participant, we counted how many transitions of the sequence were reproduced correctly. We then used the method from Rünger and Frensch (2008) to compute individual knowledge scores. The calculation of these scores takes into account how likely it is to report a certain number of transitions by mere guessing. The individual knowledge score is calculated from 100 minus the guessing probability. The score is thus adjusted for the guessing probability and shows how much the participant knows about the sequence. The participants in the blocked-SOA condition had a mean score of 92.22 (SD = 13.02). In the random-SOA condition, one participant did not complete our online survey. From the remaining 61 participants, the mean score was 90.37 (SD = 13.33). Comparably to the results from the PDWT, there was no difference between the two SOA conditions (*t*(113) = 0.74, *p* = 0.456). In addition, the Bayes Analyses for t tests confirmed the plausibility of the null hypothesis (*BF*_*01*_ = 3.93).

To sum up, the data of the post-experimental interview revealed a similar picture as the data of the PDWT: The participants in the two conditions did not differ concerning their amount of explicit sequence knowledge.

## Discussion

The goal of our study was to better understand the concrete role of RT-drops for the development of explicit knowledge in implicit sequence leaning. The initial point of the study was twofold: First, former findings have shown high correlations between RT-drop and verbal report (Haider & Frensch, 2009; Haider & Rose, 2007; Haider et al., 2011; Schwager et al., 2012). Second, the neuroimaging studies (Rose et al., 2010; Schuck et al., 2015; Wessel et al., 2012) revealed that the RT-drops were accompanied by a strong increase in neuronal activity in certain brain areas. These two findings led to the reasonable conclusion that the RT-drop reflects the point in time when a person gains explicit sequence knowledge. However, based on these correlations, it remains open whether RT-drops are a precursor for or a consequence of the generation of explicit sequence knowledge.

In the current study, we therefore focused on the manipulation of the ease of producing such an RT-drop during the SRTT training. If the RT-drops were a precursor for sequence awareness, increasing the difficulty to exhibit such an RT-drop should reduce the development of explicit knowledge. If, however, the RT-drops were a behavioral consequence of awareness, the manipulation should affect the probability of RT-drops but not the amount of explicit knowledge.

The study yielded three main results: First, task performance was affected by our manipulation. While the blocked order of the SOAs led to overall shorter RTs as well as higher error rates for the short than for the long SOAs, the random order of SOAs led to shorter reaction times with long SOAs than with short SOAs. This pattern of results is in line with the assumption that the participants in the blocked-SOA condition were better able to anticipate the onset of the next target and thus could optimize their speed of responding easier than the participants in the random-SOA condition. Second, significantly more participants in the blocked-SOA condition than in the random-SOA condition showed an RT-drop. Both findings together confirm the success of our SOA manipulation since it suggests that the participants in the random-SOA condition were less able to predict the onset of the target and, probably due to this uncertainty, showed less RT-drops. Third, despite of the finding that the two conditions differed in the number of participants who showed an RT-drop, the amount of explicit sequence knowledge was comparable in both conditions. This was also confirmed by the verbal knowledge assessed in the post-experimental interviews. Thus, our SOA manipulation did not affect the acquisition of (explicit) sequence knowledge.

Taken together, these findings complement the previous studies on RT-drops in one important point. Up till now, the high correlations between RT-drops and the amount of explicit knowledge suggested, together with the neuroimaging findings, that the RT-drop might indicate the occurrence of conscious sequence representations. Yet, since brain activity was analyzed depending on the point in time when the RT-drops appeared, it was only possible to analyze the brain activity of the participants with an RT-drop. These analyses could therefore not answer the question whether the RT-drop is the point in time when awareness occurs (as a precursor of the development of explicit knowledge) or whether it is the point in time when the participants decide to use this knowledge (as a consequence of the development of explicit knowledge). The results only showed that the brain activity was increased right before an RT-drop occurred and that the participants with an RT-drop had more explicit sequence knowledge than those without an RT-drop.

In the current study, we also found such a difference in the amount of explicit knowledge. In both conditions, more participants who showed an RT-drop developed predominantly explicit sequence knowledge than those who did not show an RT-drop. However, this correspondence was larger for the participants in the blocked-SOA condition than for those in the random-SOA condition. Thus, the current comparison of the blocked and the random-SOA conditions suggest that the emergence of a conscious representation of the sequence is, at least, partly independent of RT-drops. This independence between conscious sequence knowledge and its usage in performance allows for two alternative conclusions: First, it is conceivable that the randomly presented SOAs in the random-SOA condition increased the variability of the RTs making it harder to detect an RT-drop with our RT-drop analysis. This would be a methodological problem and would confound our results. Second, the difference in the number of participants with an RT-drop between the two conditions is reliable and reflects a result of our manipulation.

Our post hoc analyses seem to support the second conclusion. The results show that the behavioral pattern of the participants with and without RT-drop was rather similar for the two conditions. In the blocked-SOA condition as well as in the random-SOA condition, the participants who showed an RT-drop responded faster than the participants who did not. This difference increased over practice, albeit this increase was stronger in the blocked-SOA than in the random-SOA condition. However, if the varying SOAs had reduced the probability to detect an RT-drop in the random-SOA condition, we should have found a small or even no difference between these two subgroups. Thus, the results seem to be better in line with the assumption that the difference between the two conditions in the number of participants with an RT-drop is a result of our manipulation.

Regarding our main question, namely whether the RT-drop is a precursor for or a consequence of the development of explicit knowledge, our findings indicate that the RT-drops are a consequence of the development of explicit knowledge rather than a precursor. Our findings suggest that an RT-drop marks the point in time when participants rely on their explicit sequence representation and shift from stimulus-driven to plan-based performance (Haider & Rose, 2007; Hoffmann & Koch, 1997; Nattkemper & Prinz, 1997; Tubau & Lopéz-Moliner, 2004; Tubau et al., 2007). It does not seem to indicate the development of an explicit representation itself. Once developed, most of the participants in the blocked-SOA condition relied on this knowledge and used it to optimize their performance producing the sudden decrease of reaction times. In the random-SOA condition, by contrast, far lesser participants showed this sudden decrease despite having developed explicit knowledge. A possible explanation is that these participants may have gained awareness in the same manner as the participants in the blocked-SOA condition. They just were not willing or were not able to rely on this newly generated representation because the unpredictable order of the SOAs together with the short response window made this strategy rather error-prone. This might have prevented them to change the strategy from stimulus-driven to plan-based performance (Tubau & Lopéz-Moliner, 2004; Tubau et al., 2007).

This explanation seems plausible in light of other studies that investigated voluntary strategy shifts. Haider et al. (2005) showed that a switch to a new strategy is not an automatic consequence of task processing. Rather, a strategy shift occurs voluntary and controlled when the new strategy is efficient for further task performance (Gaschler et al., 2019). Further studies have shown that interfering manipulations in learning tasks often do not impair learning per se but the expression of what has been learned. In particular, impaired timing in form of, for instance, changed RSI patterns (Miyawaki, 2006; Willingham et al., 1997) or uncertainty due to random RSIs (Tubau et al., 2007), seem to influence task performance although the learning process itself seemed unaffected.

Our findings also fit well with the assumptions of the UEH (Frensch et al., 2003). According to the first assumption, we assume that in both conditions, a strong urge to respond due to the locked response window might have triggered search processes that led to the development of explicit sequence knowledge. Furthermore, the sudden decrease of reaction times, which was found in our study, is in line with the notion that sequence awareness occurs abruptly in an all-or-nothing manner. The former interpretation of the RT-drop was that it shows the point in time when explicit knowledge occurs (e.g., Rose et al., 2010). We assume that this interpretation is still correct. However, due to our results, it has to be refined by the assumption that the usage of the newly generated explicit sequence representation for performance seems to underlie voluntary control.

Despite the promising results, two limitations of our study are noteworthy. First, it was conducted online and, thus, we could not control for all confounding factors in the same manner as in lab studies. Furthermore, it was impossible for the participants to contact the investigator in the case that they did not understand the instructions. However, the wordings in our instructions have been adapted to the situation. Moreover, neither the data nor the post-experimental questionnaires gave any hints that the participants had problems to correctly perform the task. Furthermore, there already are studies in implicit sequence learning that were conducted online (e.g., Sævland & Norman, 2016) and which results were comparable to lab experiments. Lastly, potential confounding variables should have affected both the blocked-SOA and random-SOA condition.

Another limitation might be that we did not include a random block at the end of the training to assess sequence learning. Consequently, we could not distinguish sequence learning from pure practice effects on the basis of our training results. We decided not to use a random block since we were particularly interested in the relation between the expression of RT-drops and the development of explicit sequence knowledge. Such an interspersed random block bears the danger to attenuate this relation because it might hinder the participants to shift their strategy late in practice. Furthermore, the sudden decrease of RTs and the explicit sequence knowledge expressed in the PDWT cannot be explained by pure practice effects.

Finally, explicit sequence knowledge was assessed only after the training phase and we did not measure the exact point in time when the participants became aware of the sequence. Thus, it is conceivable that the RT-drop and sequence awareness developed independently. In this case, our conclusion that the RT-drop is a behavioral consequence of explicit knowledge might have been incorrect. However, Rose et al. (2010) had assessed explicit knowledge online during training. The participants were interrupted after the expression of fast responses and were asked about the reasons for these fast responses. Their findings suggest that three fast responses indicate explicit knowledge and are expressed right before an RT-drop. Taken these finding of Rose et al. (2010) into account, the conclusion, that the RT-drop is a behavioral consequence of awareness, is reasonable.

In sum, we conclude that the focus of our study on the functional role of RT-drops in implicit sequence learning and the results of the manipulation in our experiment allows the interpretation of the RT-drop as indicative for a strategy shift from stimulus-based to plan-based responding rather than the generation of an explicit knowledge representation, per se. This explicit representation comes along with the possibility to control whether one will or will not use this explicit knowledge to optimize performance.

## Data Availability

The research data will be kept saved on an external hard desk for 10 years after publishing. These data are available on request.

## Footnotes

1 Of the remaining participants, four participants in the blocked-SOA condition and five participants in the random-SOA condition missed more than 15% of the responses. Furthermore, six participants in the blocked-SOA condition and four participants in the random-SOA condition responded incorrectly in more than 15% of all trials. Due to our liberal exclusion criteria, these participants were not excluded.

2 For the purpose of our study, we decided to use this short first-order sequence. Using a more complex sequence, like, for instance, a second order sequence with more than six positions might reduce the likelihood that the participants acquire explicit knowledge about the entire sequence since the number of elements cannot be held concurrently in working memory increasing the difficulty to integrate the sequence.

3 We additionally analyzed if the RT data in the random-SOA condition differ for trials in which the SOA repeated versus changed. We found slightly longer RTs in short SOA trials when these followed a long compared to following a short SOA-trial. However, for two reasons it is rather unlikely that these slower RTs in the random-SOA condition should have affected our following RT-drop analysis. First, we median filtered the RTs in both SOA conditions with a lag of 3 subsequent trials. This median filter cancels RT-outliers and reduces noise in the data. Second, on the basis of this median filtered data, we computed the minimum functions. This implies that the values of this function changed only when the current median RT was shorter than the previous one. This minimum function then built the basis for the RT-drop analysis. Thus, the 25% of potentially slower RTs due to the long-short SOA sequences likely should not have affected the minimum functions.

## Code availability

Codes are available on request.

## References

[CR1] Cleeremans, A. (2006). Conscious and unconscious cognition: A graded, dynamic perspective. In Q. Jing, M. R. Rosenzweig, G. d’Ydewalle, H. Zhang, H.-C. Chen, & K. Zhang (Eds.). *Progress in psychological science around the world. Volume 1 neural, cognitive and developmental issues. Proceedings of the 28th international congress of psychology* (1st ed., pp. 401–418). Psychology Press. 10.4324/9780203783122

[CR2] Cleeremans, A. (2008). Consciousness: the radical plasticity thesis. In R. Banerjee & B. K. Chakrabarti (Eds.), *Models of brain and mind. Physical, computational and psychological approaches* (pp. 19–33). Elsevier. 10.1016/S0079-6123(07)68003-0 18166383

[CR3] Cleeremans A (2011). The radical plasticity thesis: How the brain learns to be conscious. Frontiers in Psychology.

[CR4] Cleeremans, A., & Jiménez, L. (2002). Implicit learning and consciousness: A graded, dynamic perspective. In R. M. French & A. Cleeremans (Eds.), *Implicit learning and consciousness: An empirical, computational and philosophical consensus in the making?* (pp. 1–40). Psychology Press.

[CR6] Dehaene S, Naccache L (2001). Towards a cognitive neuroscience of consciousness: Basic evidence and a workspace framework. Cognition.

[CR8] Del Cul A, Dehaene S, Reyes P, Bravo E, Slachevsky A (2009). Causal role of prefrontal cortex in the threshold for access to consciousness. Brain.

[CR10] Dienes Z, Seth A (2010). Gambling on the unconscious: A comparison of wagering and confidence ratings as measures of awareness in an artificial grammar task. Consciousness and Cognition.

[CR11] Esser S, Haider H (2017). The emergence of explicit knowledge in a serial reaction time task: The role of experienced fluency and strength of representation. Frontiers in Psychology.

[CR12] Esser S, Lustig C, Haider H (2021). What triggers explicit awareness in implicit sequence learning?Implications from theories of consciousness. Psychological Research.

[CR14] Frensch, P. A., Haider, H., Rünger, D., Neugebauer, U., Voigt, S., & Werg, J. (2003). The route from implicit learning to verbal expression of what has been learned: Verbal report of incidentally experienced environmental regularity. In L. Jiménez (Ed.), *Attention and implicit learning* (pp. 335–366). John Benjamins Publishing Company

[CR15] Gaschler R, Schuck NW, Reverberi C, Frensch PA, Wenke D (2019). Incidental covariation learning leading to strategy change. PLoS ONE.

[CR16] Grosjean M, Rosenbaum DA, Elsinger C (2001). Timing and reaction time. Journal of Experimental Psychology. General.

[CR21] Haider H, Eichler A, Lange T (2011). An old problem: How can we distinguish between conscious and unconscious knowledge acquired in an implicit learning task?. Consciousness and Cognition.

[CR17] Haider H, Frensch PA (2005). The generation of conscious awareness in an incidental learning situation. Psychological Research Psychologische Forschung.

[CR18] Haider H, Frensch PA (2009). Conflicts between expected and actually performed behavior lead to verbal report of incidentally acquired sequential knowledge. Psychological Research Psychologische Forschung.

[CR20] Haider H, Frensch PA, Joram D (2005). Are strategy shifts caused by data-driven processes or by voluntary processes?. Consciousness and Cognition.

[CR19] Haider H, Rose M (2007). How to investigate insight: A proposal. Methods.

[CR22] Hoffmann J, Koch I (1997). Stimulus-response compatibility and sequential learning in the serial reaction time task. Psychological Research Psychologische Forschung.

[CR23] JASP Team (2020). JASP (Version 0.14.1) [Computer software]

[CR300] Jeffreys H (1961). The Theory of Probability.

[CR24] Koch I (2007). Anticipatory response control in motor sequence learning: Evidence from stimulus–response compatibility. Human Movement Science.

[CR25] Koriat A (2012). The self-consistency model of subjective confidence. Psychological Review.

[CR28] Lawson RR, Gayle JO, Wheaton LA (2017). Novel behavioral indicator of explicit awareness reveals temporal course of frontoparietal neural network facilitation during motor learning. PLoS ONE.

[CR29] Marti S, Dehaene S (2017). Discrete and continuous mechanisms of temporal selection in rapid visual streams. Nature Communications.

[CR30] Miyawaki K (2006). The influence of the response-stimulus interval on implicit and explicit learning of stimulus sequence. Psychological Research Psychologische Forschung.

[CR31] Morey, R. D., & Rouder, J. N. (2015). BayesFactor (Version 0.9.11–3) [Computer software]

[CR32] Nattkemper D, Prinz W (1997). Stimulus and response anticipation in a serial reaction task. Psychological Research Psychologische Forschung.

[CR33] Niemi P, Näätänen R (1981). Foreperiod and simple reaction time. Psychological Bulletin.

[CR34] Nissen MJ, Bullemer P (1987). Attentional requirements of learning: Evidence from performance measures. Cognitive Psychology.

[CR35] Pavlovia. https://pavlovia.org/. Accessed 21 July 2020.

[CR36] Persaud N, McLeod P, Cowey A (2007). Post-decision wagering objectively measures awareness. Nature Neuroscience.

[CR37] Prolific | Online participant recruitment for surveys and market research. https://www.prolific.co/. Accessed 21 July 2020.

[CR38] Rose M, Haider H, Büchel C (2010). The emergence of explicit memory during learning. Cerebral Cortex.

[CR40] Rouder JN, Morey RD, Speckman PL, Province JM (2012). Default Bayes factors for ANOVA designs. Journal of Mathematical Psychology..

[CR39] Rouder JN, Speckman PL, Sun D, Morey RD, Iverson G (2009). Bayesian t tests for accepting and rejecting the null hypothesis. Psychonomic Bulletin & Review.

[CR41] Rünger D, Frensch PA (2008). How incidental sequence learning creates reportable knowledge: The role of unexpected events. Journal of Experimental Psychology: Learning, Memory, and Cognition.

[CR42] Sævland W, Norman E (2016). Studying different tasks of implicit learning across multiple test sessions conducted on the web. Frontiers in Psychology.

[CR43] Schlaghecken F, Sturmer B, Eimer M (2000). Chunking processes in the learning of event sequences: Electrophysiological indicators. Memory & Cognition.

[CR44] Schuck NW, Gaschler R, Wenke D, Heinzle J, Frensch PA, Haynes J-D, Reverberi C (2015). Medial prefrontal cortex predicts internally driven strategy shifts. Neuron.

[CR45] Schwager S, Rünger D, Gaschler R, Frensch PA (2012). Data-driven sequence learning or search: What are the prerequisites for the generation of explicit sequence knowledge?. Advances in Cognitive Psychology.

[CR301] Shanks DR, Lamberts K, Goldstone R (2005). Implicit learning. Handbook of cognition.

[CR47] Shin JC (2008). The procedural learning of action order is independent of temporal learning. Psychological Research Psychologische Forschung.

[CR48] SoSci Survey GmbH. https://www.soscisurvey.de. Accessed 21 July 2020.

[CR49] Tubau E, Lopéz-Moliner J (2004). Spatial interference and response control in sequence learning: The role of explicit knowledge. Psychological Research Psychologische Forschung.

[CR50] Tubau E, Lopéz-Moliner J, Hommel B (2007). Modes of executive control in sequence learning: From stimulus-based to plan-based control. Journal of Experimental Psychology: General.

[CR51] Wessel JR, Haider H, Rose M (2012). The transition from implicit to explicit representations in incidental learning situations: More evidence from high-frequency EEG coupling. Experimental Brain Research.

[CR52] Whittlesea BWA (2004). The perception of integrality: Remembering through the validation of expectation. Journal of Experimental Psychology: Learning, Memory, and Cognition.

[CR53] Willingham DB, Greenberg AR, Cannon T (1997). Response-to-stimulus interval does not affect implicit motor sequence learning, but does affect performance. Memory & Cognition.

[CR54] Zacks JM, Speer NK, Swallow KM, Braver TS, Reynolds JR (2007). Event perception: A mind/brain perspective. Psychological Bulletin.

